# Supplementary material for: Seminoma and Embryonal Carcinoma Footprints Identified by Analysis of Integrated Genome-Wide Epigenetic and Expression Profiles of Germ Cell Cancer Cell Lines
Source: PLoS One. 2014 Jun 2;9(6):e98330. doi: 10.1371/journal.pone.0098330 (PMC4041891; doi:10.1371/journal.pone.0098330)
Supplement: File S1 — ZIP file containing DMRforPairs output for significant regions. Please start from the html files. (ZIP) [file pone.0098330.s008.zip › figures/1817.pdf]

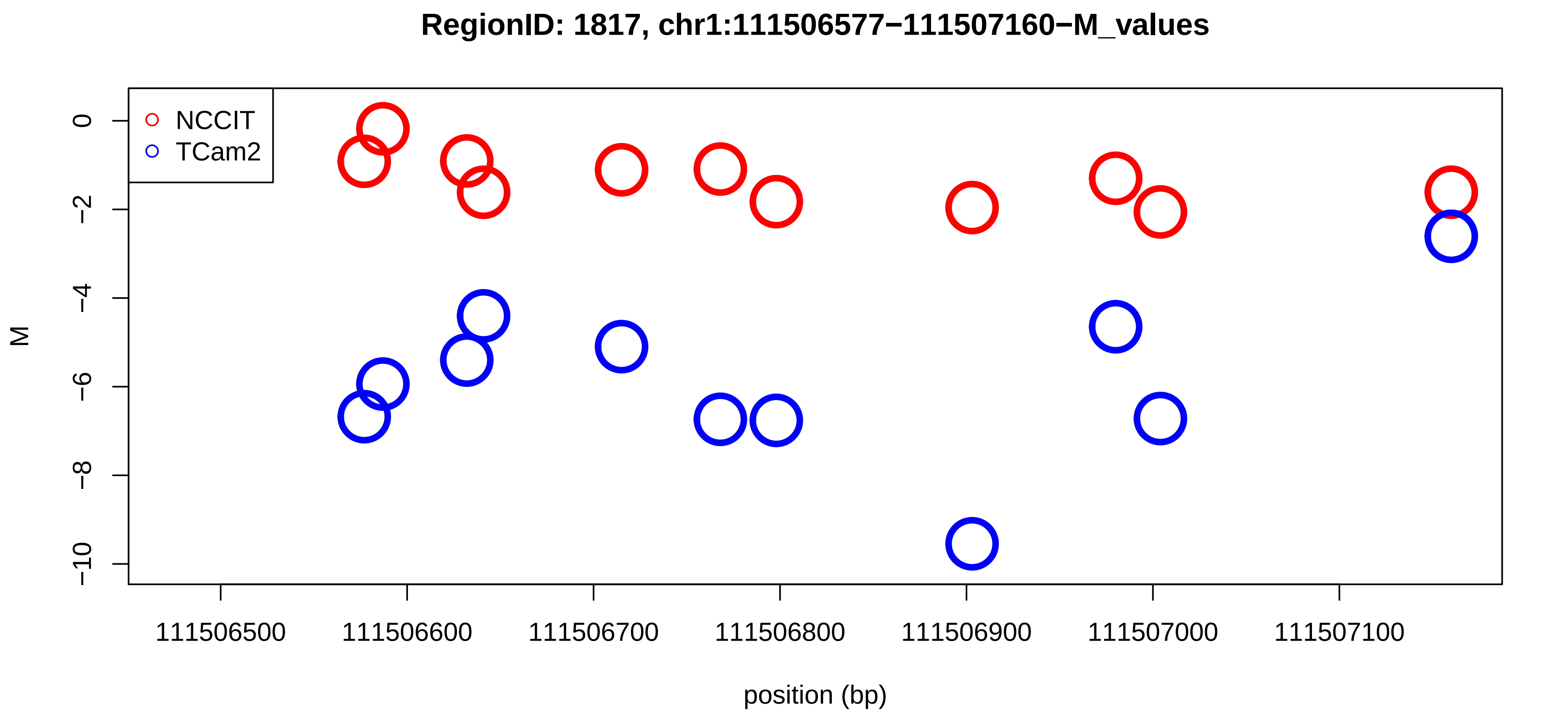

RegionID: 1817, chr1:111506577−111507160−M_values
NCCIT
TCam2
M
0
−2
−4
−6
−8
−10
111506500
111506600
111506700
111506800
111506900
111507000
111507100
position (bp)


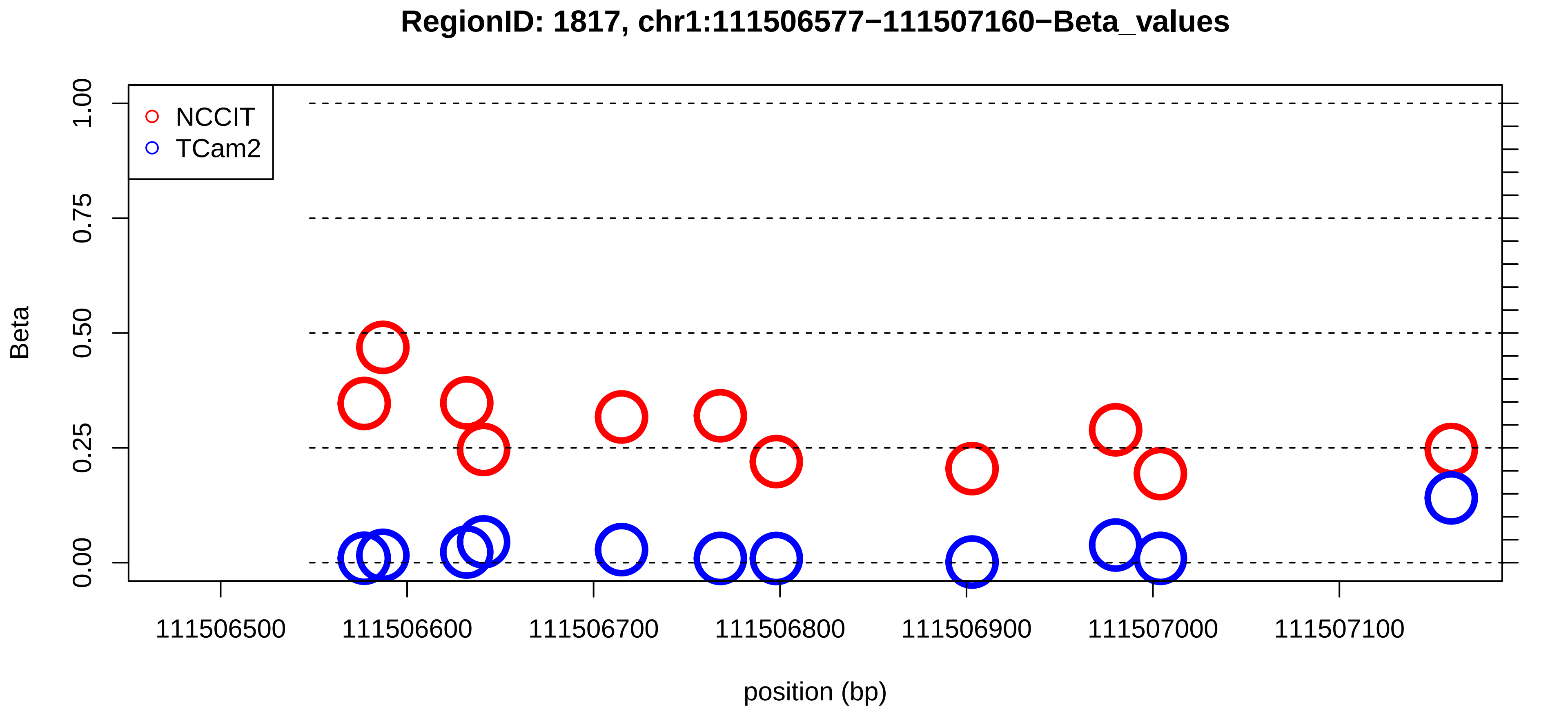

RegionID: 1817, chr1:111506577−111507160−Beta_values
NCCIT
TCam2
Beta
1.00
0.75
0.50
0.25
0.00
111506500
111506600
111506700
111506800
111506900
111507000
111507100
position (bp)

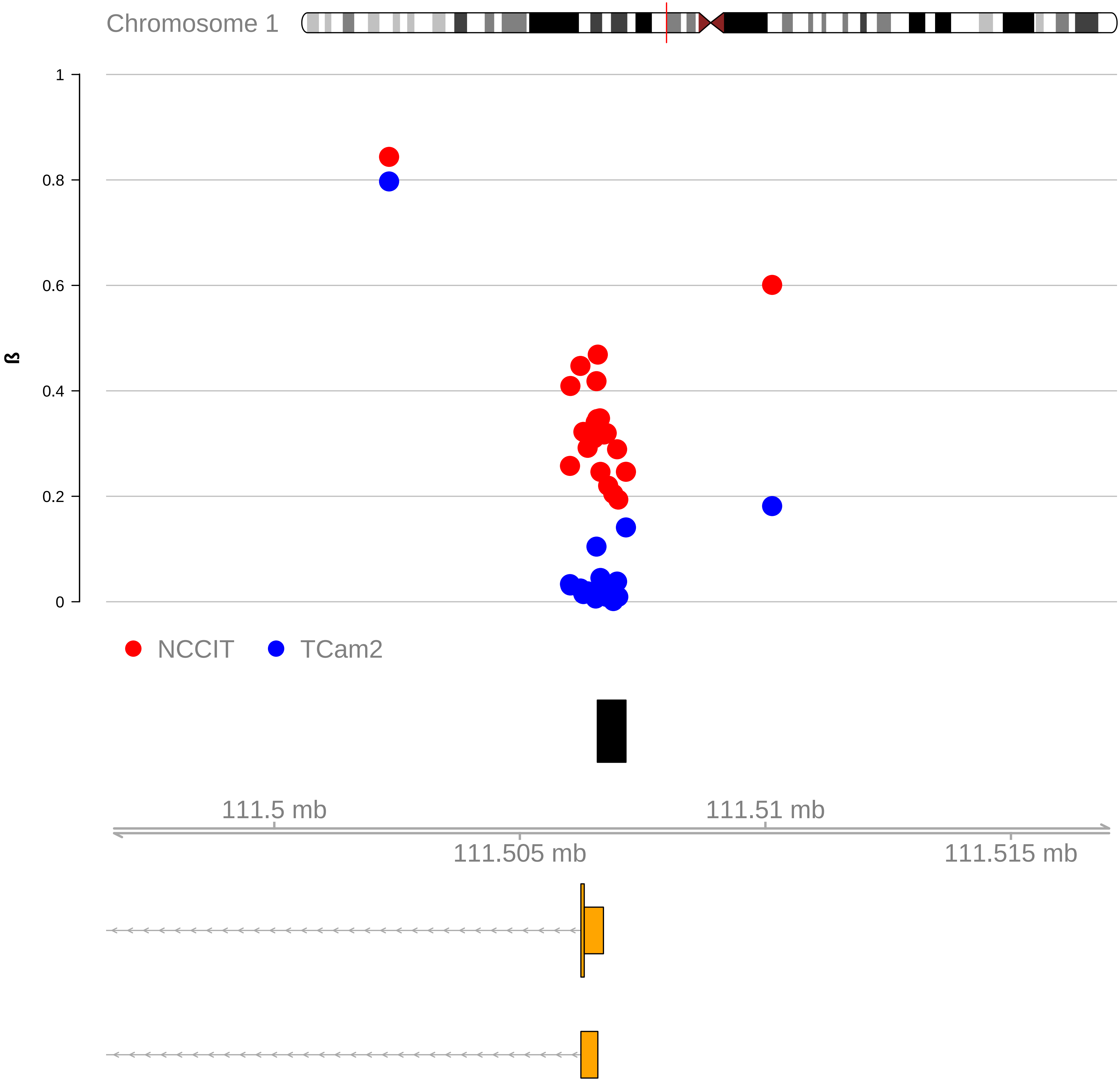

Chromosome 1
ß
1
0.8
0.6
0.4
0.2
0
NCCIT
TCam2
111.5 mb
111.51 mb
111.505 mb
111.515 mb
